# Interprofessional education and collaboration between general practitioner trainees and practice nurses in providing chronic care; a qualitative study

**DOI:** 10.1186/s12909-020-02206-1

**Published:** 2020-09-03

**Authors:** R. van der Gulden, N. D. Scherpbier-de Haan, C. M. Greijn, N. Looman, F. Tromp, P. W. Dielissen

**Affiliations:** Department of Primary and Community care, Radboud university medical centre, P.O. Box 9101, 6500 HB Nijmegen, The Netherlands

**Keywords:** Interprofessional education, Interprofessional collaboration, Chronic care, GP training practice, GP training program

## Abstract

**Background:**

Effective interprofessional collaboration (IPC) is essential for the delivery of chronic care. Interprofessional education (IPE) can help support IPC skills. This makes IPE interesting for GP practices where chronic care is delivered by GPs together with practice nurses, especially for GP trainees who have to learn to collaborate with practice nurses during their training. The aim of this study is to gain insights in how IPE and IPC occur between GP trainees and practice nurses during the delivery of chronic care in GP training practices.

**Methods:**

We conducted a qualitative research using semi structured focus groups and interviews with GP trainees, practice nurses and GP supervisors. All respondents were primed to the subject of IPE as they had followed an interprofessional training on patient-centred communication. The verbatim transcripts of the focus groups and interviews were analysed using thematic analysis.

**Results:**

Despite the overall positive attitude displayed by respondents towards IPE and IPC, the occurrence of IPE and IPC in GP training practices was limited. Possible explanations for this are impeding factors such as limited knowledge, prejudice, lack of role models and a hierarchical organisational structure. Contributing to IPE and IPC use was the integration of IPE in daily practice, e.g. via recurring scheduled meetings.

**Conclusion:**

We found a limited occurrence of IPE and IPC in GP training practices. Our results show a discrepancy between respondents enthusiasm for IPE and IPC and their actual behaviour. IPE activities have to be initiated in GP training practices, otherwise, despite good intentions, IPE and IPC will be ineffective.

## Background

Well attuned collaboration between professionals is key in the quality of chronic care [1]. Chronic care delivered in primary care settings - for patients with diabetes mellitus, chronic obstructive pulmonary disease, asthma, cardiovascular disease and prevention - is considered complex [2]. The high level of multimorbidity in chronic care with interactions between diseases leads to clinical complexity [3]. In addition, the fact that one patients sees many different healthcare professionals can easily lead to fragmentation of care [4, 5].

In this complexity, effective interprofessional collaboration (IPC) between general practitioner (GP) and practice nurse is essential to provide high quality care, as practice nurses are (in the Netherlands) involved in more than 88% of chronic cases [6]. To make this collaboration work, GPs and practice nurses require interprofessional collaborative competencies. A shared understanding of the concept of care and of each other’s role and expertise is essential [7]. This allows GPs to share responsibilities with practice nurses. Studies show that collaboration is enhanced when GPs actively seek for clarification and understanding of the practice nurses’ role and expertise [8, 9]. However, many GPs have limited knowledge of the role and expertise of their practice nurses, and practice nurses often see their role as a GP’s assistant [9]. This limits the nurses’ role within the team and thus inhibiting effective IPC between GP and practice nurses.

Interprofessional education (IPE) can help healthcare professionals become competent interprofessional collaborators [10]. IPE can support professionals in developing their IPC skills [10]. IPE occurs when two or more different professions learn with, from and about one another [11, 12]. In previous years IPE has received growing scientific attention. Several studies systematically reviewed the effects of IPE [10] and the barriers and enablers when implementing IPE [13]. These studies show that IPE is often positively evaluated by students and increases collaborative knowledge and skills [10].

However, the implementation of IPE does not occur automatically. Limited understanding about the norms and practices of other health professions forms a barrier [14]. Healthcare staff should be role models by showing effective IPC to their students [15], but this is not always the case. Other reported barriers to the implementation of IPE are professional hierarchy and lack of time [8, 16].

We have discussed the importance of effective IPC between GPs and practice nurses in chronic care. Considering this, it is essential that GP trainees engage in IPC with practice nurses during their training, as this is an important skill for their future profession. Consequently, IPE between GP trainees and practice nurses may be of value, especially in light of the difficulties that exist concerning IPC between GPs and practice nurses [1, 9]. Given the proximity between GP supervisors, GP trainees and practice nurses, it could be assumed that IPE and/or IPC take place to a certain extent within GP training practices. However, to our knowledge, no studies have yet focused on IPE and IPC between GP trainees and practice nurses.

The aim of this study was to gain insight into how IPE and IPC occur in GP training practices. With this knowledge we can improve the conditions for implementing IPE in GP training programs, to make sure that future GPs are capable of effective IPC with practice nurses. We formulated the following primary research questions with respect to IPE and IPC in chronic care between GP trainees and practice nurses in GP training practices:

1. To what extent do GP trainees and practice nurses engage in IPE and IPC and what are their experiences?
2. What are the contributing and impeding factors for IPE and IPC between GP trainees and practice nurses?

## Methods

### Context

In the Netherlands, it is common that multiple GP practices cooperate in regional partnerships with the aim of improving chronic care. Affiliated practices exchange knowledge and services, for example, via interprofessional meetings where chronic health related topics are discussed.

This study was conducted by staff members at Radboud University Medical Centre’s GP specialty training in cooperation with a regional partnership called *De ondernemende Huisarts* (DOH) which translates as the ‘entrepreneurial GP’. The DOH provides training on patient-centred communication in chronic care to encourage IPE and IPC between GPs, GP trainees, and practice nurses (see Table 1 for the goals and learning activities of this training program).

**Table 1:** Goals and learning activities of training on patient-centred communication provided by the DOH

**Goals:** 1. Participants learn that in providing personalized chronic patient care, interprofessional education and collaboration is essential, e.g. development of teamwork skills and appreciation of each profession’s scope and role. 2. Participants learn patient-centred communication for the provision of chronic care in primary care setting. 3. GP trainees are challenged with the idea that not only their GP supervisor but also nurse practitioners can be their teachers and can be consulted for advice. **Learning activities in interprofessional meetings:** 1. Lecture on patient-centred communication in chronic care. 2. Lecture on IPC and IPE. 3. Video feedback session to train communication skills. 4. Discussion sessions led by communication and collaboration experts. **Learning activities in GP training practices:** 1. Direct observation of each other’s consultations (GP trainee, nurse practitioner and GP) using an assessment instrument on patient-centred communication to provide feedback. 2. Scheduled case consultations between GP trainee - nurse practitioner - GP to discuss communication and treatment. 3. GP trainee and practice nurse performing consultations together.	

### Reflexivity statement

The study was performed by a research team with different professional disciplines and backgrounds: psychology (RG, FT, NL), cultural anthropology (CMG) and medicine (PD, NS). This provided the opportunity to combine and connect knowledge and expertise from different fields for the purpose of this study [17]. As mentioned, all authors are affiliated with the GP speciality training program and two of them (PD, NS) also work as GP, and therefore have a professional understanding of GP work. This may have led to an overrepresentation of the view of the GP (trainee) compared to that of the practice nurse.

### Design

We took a qualitative study approach because of the explorative nature of the research question. We chose focus groups as the primary method of data collection because they best facilitate an exploration of the range of perspectives within and between different groups [18]. Even though, GP trainees and practice nurses were our primary groups of interest we also decided to include GP supervisors in our study. We expected that the latter could provide us with (additional) information, as they mentor the GP trainee and work closely with the practice nurse. Moreover, bearing in mind the importance of role models within IPE we considered the opinions of GP supervisors of value. In order to ensure safety and to provide the conditions for respondents to speak freely, we composed homogenous focus groups, where GP supervisors, GP trainees and practice nurses could participate separately.

Due to our respondents’ workload, in many cases they were unable to join one of our focus groups. As a result we could only plan three focus groups. We expected that this would not be sufficient to reach saturation, so, we conducted additional interviews by telephone in order to collect sufficient data. This provided us with an opportunity to enrich the data and meet the standard of data triangulation [19].

### Procedure

All GP supervisors (*N* = 23), GP trainees (*N* = 21), and practice nurses (*N* = 17) of the GP training practices affiliated with the DOH, that participated in the patient-centred communication training could be included. We invited 33 GP supervisors, 39 GP trainees and 45 practice nurses. Respondents were selected through convenience sampling. All participants were invited via email to participate in a focus group. A letter of information and a consent form were attached to this email. As soon as we anticipated that we would not be able to conduct enough focus groups, we sent a reminder to non-responders in which we offered the opportunity to be interviewed by telephone at a time of their choice. Together, this resulted in the participation of nine GP supervisors, twelve GP trainees and sixteen practice nurses. All GP supervisors had more than 5 years’ experience in training GP trainees. Table 2 shows the characteristics of the respondents.

**Table 2:** Descriptive characteristics of the respondents. NA = not available

	GP supervisor	GP trainee	Practice nurse
**Number of respondents**	9	12	16
**Participation via**			
**Focus group**	0	3	10 (4, 6)
**Interview**	9	9	6
**Sex**			
**Female**	4	9	16
**Male**	5	3	0
**Age (min-max)**	48 - 66 years	27 - 32 years	NA

At the start of the focus groups the consent form, which was sent in advance, was signed by the respondents, during the telephone interviews consent was given verbally. The focus group was held for approximately one hour, the telephone interviews lasted about half an hour. Respondents received a gift cheque worth 50 Euros for their participation.

All focus groups and interviews were conducted in Dutch by two interviewers at a time. The interview pairs varied (RG, FT, PD, CMG, NL). The focus groups took place in a private office room at our GP training location in the Radboud university medical centre in Eindhoven. A semi-structured interview guide was used to conduct the focus groups and interviews (Additional file 1). This guide was initially designed during an expert meeting, which involved stakeholders from the GP training institute and the DOH. The guide evolved over time as insights about IPE and IPC in GP training practices grew; the initial interview guide and the need for adjustments was discussed regularly between the researchers. The focus groups and interviews were conducted between November 2016 and October 2017.

The focus groups and interviews were audio recorded. Verbatim transcripts of the recordings were made by one of the researchers (RG), names and other personal data were not transferred to the transcripts. All analysis was in Dutch. The selected quotes for the manuscript have been translated by two authors (RG, FT). Differences in opinion about the possible loss of meaning of the initial translation were discussed between the other authors.

### Analysis

Analysis of the verbatim transcripts started after the first focus groups, and was thus performed simultaneously with data collection. This iterative manner of working allowed us to use insights from data analysis in subsequent focus groups and interviews. The analysis started with deductive coding, thereby using the different sub-questions of the research questions as guidance:

- 1a. To what extent do GP trainees and practice nurses engage in IPE and IPC?
- 1b. What are their experiences?
- 2a. What are the contributing factors for IPE and IPC between GP trainees and practice nurses?
- 2b. What are the impeding factors for IPE and IPC between GP trainees and practice nurses?

During the coding process the initial sub-questions were refined and different codes belonging to the sub-questions emerged. Any need for adapting the coding scheme was discussed by the research team (RG, FT, CMG, PD). During coding of the final interviews, no new codes arose, which indicated that thematic data saturation had been achieved. After all the transcripts were coded, we searched for transcending themes and how they interacted. Atlas.ti was used to organise data during analysis.

## Results

The codes and themes that surfaced during analysis are reported in line with the research questions and are shown in Table 3.

**Table 3:** The codes and themes that emerged during data-analysis, which was deducted on basis of the different sub-questions of the research questions

Sub-questions	Codes	Themes
Topics discussed during IPE and IPC between GP trainee and practice nurse	-Pulmonary function tests -Insulin therapy -Interpretation laboratory tests -Organisation of chronic care -Exploring patients’ perspective -Structuring the consultation -Motivational interviewing	Knowledge and skills concerning chronic care Communication skills
Sentiments of GP trainee and practice nurse concerning IPE and IPC	- Enthusiasm - Inspiration	Positive outlook
Contributing factors for IPE and IPC	-Scheduled IPE/IPC activities -Assigning tasks and responsibilities	Integration of IPE/IPC in the organisation
Impeding factors for IPE and IPC	-Opinions on methods used by the other profession -Lack of knowledge of the others’ professions -Experiencing less necessity -Not taking any initiative -Organisational structure of the GP training practice -Direction and frequency of communication between the triad -Hierarchy -Behaviour and opinions of GP supervisor concerning IPE/IPC -IPE is not facilitated	Prejudice Lack of urgency Hierarchy Lack of role models

### To what extent do GP trainees and practice nurses engage in IPE and IPC and what are their experiences?

When asked for their opinion about IPE and IPC, both GP trainees and practice nurses reacted with great enthusiasm. They said that IPE and IPC are important and some went even further by stating that they are a necessity. When asked to give a definition of IPE, many practice nurses mentioned the opportunity to learn from another profession as did the GP trainees. GP trainees also saw an opportunity to learn about the profession of the practice nurse.

The experience with IPE was, for many respondents, limited to their participation in the training provided by the DOH. During the training program activities, GP trainees stated that the practice nurses could provide them with medical information and skills needed for the provision of chronic care, such as pulmonary function tests and measures to avoid glucose fluctuations in diabetic patients. GP trainees reported that, by observing practice nurses, they gained insights in the tasks, role and responsibilities of the practice nurse.

*GP trainee 4: I also enjoy gaining better insights into the organisation of chronic care: how often should certain things be checked, what to do when we find something atypical, when do you follow up, and whose responsibility is that, practice nurse or GP?*

Some of the GP trainees mentioned that they were able to learn from the practice nurse about various communication skills, such as motivational interviewing. In contrast, others stated that their communication skills were superior to those of the practice nurses, and therefore saw no learning opportunities in this respect.

Although, practice nurses said they learned from the GP trainees during shared meetings they found it hard to formulate concrete examples of what they had learned. The acquisition of state-of-the-art knowledge from GP trainees was mentioned by practice nurses. This included not only medical knowledge, but also working with computers and other technical devices. Moreover, they said that working with GP trainees was inspiring.

*Practice Nurse 7: ..a GP trainee has less cut-and-dried solutions than GPs, is more inclined to say “Wait a minute.. let’s search for an answer”. That’s really nice, because you also learn new ways of finding information.*

### What are the contributing and impeding factors for IPE and IPC between GP trainees and practice nurses?

#### Contributing factors

Respondents of GP training practices that actively engaged in IPE reported examples of IPE and IPC activities that were integrated into their working routine. For example, through scheduled weekly meetings between different professionals regarding patient cases or specific themes. These respondents usually reported that in their practice someone was responsible for the organisation and planning these type of meetings.

*GP trainee 2: Well, what we do, we have meetings almost every day with the practice nurses, and then we discuss nearly every patient she has seen that day. We did that with the three of us, so my supervisor, the practice nurse, and me. Firstly she brought up something “I’ve seen this patient and this and that was going on, these are his labs..” and eh.. then we looked together at it.. or I looked at the results. And if there were any irregularities we would go deeper into that.*

*GP supervisor 6: Hmm.. interprofessional education, well, I find that.. We just try to work interprofessionally, so to speak. Regarding a patient there’s the assistant, two or three GPs [..] and the practice nurse. We try to make the connections between them as much as possible. So when something comes up and colleague or another discipline needs to know something about it, we try to inform each other. That’s essentially the route where a lot of learning takes place.*

#### Impeding factors

##### Prejudice

During our interviews and focus groups GP supervisors, GP trainees, and practice nurses mentioned preconceived ideas they had about each other. For instance, all parties expressed opinions on the balance between adhering to good practice guidelines, and paying attention to the patient’s personal needs and possibilities.

*Practice nurse 15: And the other thing that I notice with some of them [GP trainees], is that they don’t have a broad enough view, like they’re wearing blinkers. When data are available, they only look at that data to come to a conclusion. While the story of the patient is often at least half of the treatment you end up with.*

*GP trainee 1: Well, sometimes there are topics that are really important for the patient.. [...] that’s difficult because of all the other things that they [practice nurses] need to discuss are according to protocol, but sometimes you just need to discuss something that is not related to data or lab results, so to say.*

*GP supervisor 4: What is important for us is that we can see, at least once, how the practice nurse does her consultation. That was a real eye-opener to me, just following the protocol, and paying little attention for the patient’s personal story.*

Notably, some respondents expressed uncertainty about the exact tasks and responsibilities of the other party. This potentially fosters the abovementioned prejudice. This kind of prejudice might be fuelled by a lack of knowledge about each other’s profession.

*Practice nurse 2: Well I actually don’t really know about eh.. the content of their specialty training and what the supervision entails and so on.*

##### Lack of urgency

The respondents uttered little need to change their routines and integrate IPE in their working practices, as they managed well without IPE.

*Practice nurse 3: So in that way it [IPE] is... it could come in handy. But if I follow my own approach I also manage.*

*Interviewer 2: Would you like to do more with it [IPE]?*

*Practice nurse 2: No, I actually believe it’s fine as it is right now.*

*GP trainee 3: So I think it can be useful to a certain extent. But you have to ask yourself, since you lose ‘valuable working time’, to what extent that weighs up. So firstly it should appear to be worthwhile.*

In addition, multiple respondents expressed that they waited for the other party to initiate IPC.

*Practice nurse 13: I leave that up to them. Because they can always join me, that’s never a problem for me. [..] they can just come by my office without any hesitation. But they’re always busy. So it doesn’t happen in actual practice.*

*Practice nurse 2: For some kind of reason it doesn’t happen. I always have something like, if I then.. if I have a question I just ask the GP when I spot them in the hallway, or something.*

##### Organisational structure of GP training practices

**Fig. 1:**
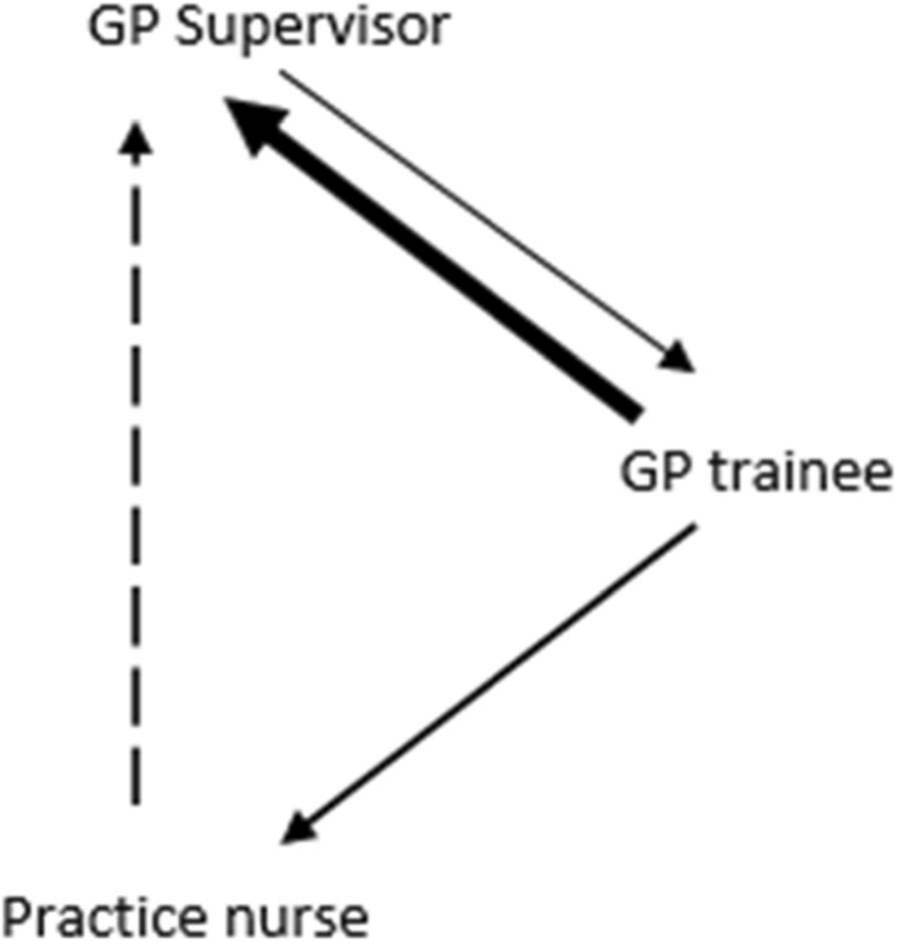
Direction (the arrow indicates who consults who) and frequency (thicker lines indicate higher frequency) of communication between GP supervisor, GP trainee and practice nurse.

GP training practices are led by the GP supervisor, which means that practice nurses and GP trainees are accountable to the GP supervisor. This organisational structure is one factor in explaining the direction and frequency of the communication patterns within the practice that surfaced during the focus groups and interviews (see Fig. 1):

- GP supervisors and GP trainees both mentioned that they interact intensively during daily meetings. During these interactions, the GP trainee generally consults the GP supervisor, but some GP supervisors also mentioned possibilities to learn from their trainee.
- GP trainees approach practice nurses with specific questions regarding chronic care. In general, the practice nurse does not initiate contact with the GP trainee.
- Practices nurses infrequently approach GP supervisors with questions. In the interviews with the GP supervisors, we neither heard nor asked whether GP supervisors consulted their practice nurses.

Practice nurses and GP trainees mention that there are, in general, egalitarian professional relationships within the practice. However, the direction and frequency of communication shows another picture. Indications of the presence of existing hierarchy surfaced during interviews and focus groups.

*Practice nurse 12: Well, I think we’re all here for the same purpose, so we’re all equal, I think. And … I guess there’s some room for improvement there, so to speak. I don’t think that with regard to the GP trainees, but with, with the others, a bit older GPs well, their ego gets in the way sometimes.*

*Interviewer 1: So, that’s a team of equal partners?*

*GP trainee 11: Yes, well.. yes, everybody brings their own.. own knowledge. You see, it’s of course the partnership of the GPs, they’re.. they employ the practice nurses [...] There’s.. still.. a little difference, because.. well, you’re a GP and that’s a bit of a different position.*

*GP trainee 5: [Explains the collaboration with a geriatrician] your level of thought aligns you know, you can complement each other. With a practice nurse you feel like you might, that doesn’t have to be like that, but.. well..*

##### Lack of role models

As mentioned, GP supervisors were included in the study considering their potential to function as role models. However, in Fig. 1 it is clear that there is little contact between the GP supervisor and practice nurse, showing that GP supervisors do not actively engage in IPC with the practice nurse. During the interviews some GP supervisors also expressed little necessity for IPE with the practice nurse.

*GP supervisor 1: Well, I can imagine that the need for IPE is bigger for some than for others. The need of others I can’t judge, but for me.. I am an older GP, and I notice I don’t feel the need for that. Partly because I already teach a GP trainee*

*GP supervisor 8: Well, it’s mainly something for the practice nurse and GP trainee, well euh, how do I say this, should be their responsibility. … it is more with regard to their own learning process.*

In addition, GP supervisors did not seem to facilitate IPE within their practices, as it was often mentioned that there were problems concerning the organisation of IPE. This was partly because respondents perceived the other party to be responsible for collaboration and also because IPE was perceived as time-consuming and it interfered with daily tasks and responsibilities.

## Discussion

This qualitative study aimed to gain insights in IPE and IPC between GP trainees and practice nurses working in GP training practices. Our results showed that the IPE experiences of our respondents were mainly confined to the training on patient-centred communication they participated in. In general, IPC between the two professions was limited, as GP trainees and practice nurses seemed to work alongside each other and in many cases only contacted each other when specific questions arose. This lack of engagement in IPE and IPC contrasted with the positive outlook respondents expressed. This raises the question why they do not pursue IPE and IPC more actively if they have such a positive attitude about it.

We found a number of impeding factors that may explain this discrepancy between attitude and behaviour. Respondents seemed unaware of the various ways in which they could participate in IPE, which might have contributed to a lack of initiative regarding to their approach the other party. Another impeding factor that surfaced during the interviews, aligns with that noted in the existing literature and concerns limited knowledge about each other’s profession [14]. This lack of understanding seemed to contribute to prejudiced ideas that existed between the two professions. Prejudice can also be fuelled by hierarchy [20], which already has been mentioned as barrier to IPE [8]. We also found indications that hierarchy within the training practices interfered with IPE and IPC between our respondents.

In addition, the interviews indicated that the two professions differed in their work philosophies and ethics. For example, GP trainees, who are trained to conduct highly structured ten minute consultations saw little value in longer consultations, as they feel like this leads to ‘unstructured’ conversations. In contrast, practice nurses had the idea that these ten minutes consultations failed to provide patients with the attention needed. This finding fits with research that shows that groups of professionals form their own culture, consisting of unique habits, customs and language [21]. These professional cultures can lead to difficulties with interprofessional teamwork, via prejudice, differing values and norms and the use of occupational specific terms and language [22]. Professional cultures are also associated with a reluctance to change [23], which could explain the lack of urgency of respondents to change current routines in order to engage in IPE and IPC.

The GP supervisors appear to contribute to this lack of urgency. They show little necessity to engage in IPE with the practice nurse themselves, as the interaction between the GP supervisor and practice nurses is limited. In addition, IPE activities are not actively facilitated by GP supervisors, e.g. GP trainees and practice nurses do not experience the time required to engage in IPE. Thereby, GP supervisors do not function as positive role models, which is considered essential for the occurrence of IPE [15].

**Fig. 2:**
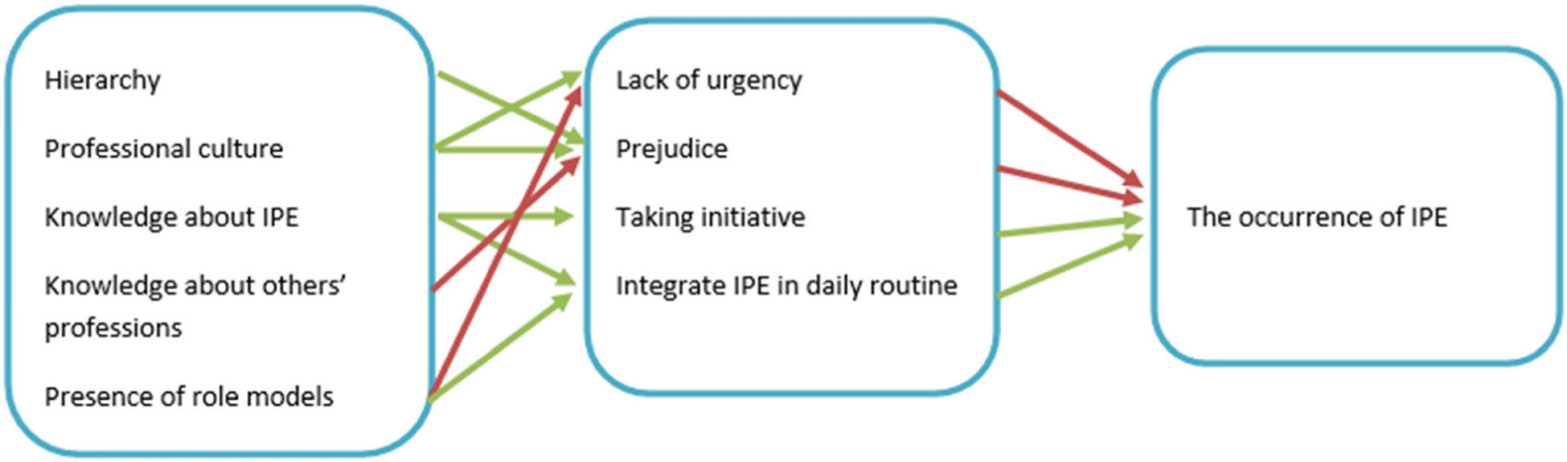
Overview of different factors related to the occurrence of IPE in GP training practices. Green arrows indicate a positive relationship between the factors, where red arrows indicate a negative relationship between different factors.

In comparison to the impeding factors, respondents did not mention many contributory factors. All examples of well attuned IPE en IPC were found in GP training practises where IPE and IPC was integrated in daily practice routines. Recurring meetings between different professionals, often focusing on specific themes or patient cases, were structurally scheduled. In these cases it was clear who was responsible for the planning of these meetings. This aligns with research that indicates that professional activities at an organisational level are necessary to achieve successful IPE and IPC [24]. Figure 2 presents an overview of these findings.

This model can be seen in the light of the theory about communities of practice [25, 26]. Communities of practice are defined as *groups of people who share a concern, a set of problems, or a passion about a topic, and who deepen their knowledge and expertise in this area by interacting on an ongoing basis* [27]. This tradition of research already shows that bringing a group of professionals together, does not necessarily mean that learning will take place [28]. Consequently, interventions on the contributing and impeding factors mentioned in Fig. 2 are needed, if we want to exploit the potential of these mini-communities of practice for IPE.

### Implications for practice and future research

Our study underlines that IPE and IPC does not happen automatically when different health professionals care for the same patients. Considering Fig. 2, there are a number of factors which can help to promote IPE and IPC between different health professionals:

1. Inform all professionals involved in each other’s profession.
2. Inform all professionals involved in (the importance of) IPE and IPC.
3. Challenge (senior) staff members to lead by example.
4. Make IPE activities part of the working routine, instead of adding these to regular tasks and responsibilities.
5. Ensure that all involved understand their responsibilities concerning the IPE activities.

Future research on IPE in general practice should consider the work routines of professionals in the workplace, before an intervention is launched, as our research showed the importance of integrating IPE in the working routine. Ideally, a cluster randomised trial with intervention and ‘usual education’ practices would reveal patient reported outcomes. However, it would be hard to control for confounding factors.

### Limitations

Our study was conducted in a limited number of GP training practices in the south of the Netherlands. Generalisability of the results can therefore be questioned, although our results are comparable with the current literature on IPE and IPC.

In addition, the connection between the study and the DOH training program, seemed to have primed respondents to confine IPE to the purpose of patient-centred communication, as this was the program’s core topic. During the focus groups and interviews we tried to broaden the views of respondents with our line of questioning.

The quotes of participants were translated by two of the authors, who are not native speakers. This may have caused a subtle loss of detail. Their translations however, have been checked by a professional translator.

In this study, we focused on IPE and IPC between GP trainee and practice nurse as they are important for the delivery of chronic primary care. However, the collaborative playing field is far more complex than our research entailed, and include a variety of different professionals that extend outside the GP practice.

Finally, the decision to make use of interviews in addition to focus groups led to the loss of information concerning group interaction.

## Conclusion

The occurrence of IPE and IPC between practice nurses and GP trainees in GP training practices is still limited. There is a discrepancy between expressed enthusiasm for IPE and actual behaviour. This study found a number impeding factors that can explain this difference: limited knowledge concerning IPE and each other, a lack of role models, a low level of experienced urgency, the existence of prejudice and a hierarchical organisational structure. Contributing to the occurrence of IPE and IPC was the integration of IPE in daily practice, e.g. by recurring scheduled meetings between different professionals. For this to happen there needs to be clarity about the division of tasks and responsibilities concerning IPE.

## Supplementary information

**Additional file 1.** Final version of the interview guide.

## Data Availability

The anonymized datasets used and/or analysed during the current study are available from the corresponding author on reasonable request.

## Abbreviations

DOH: *De ondernemende huisarts* which translates as the ‘entrepreneurial GP’
GP: General practitioner
IPC: Interprofessional collaboration
IPE: Interprofessional education
