# Supplementary material for: Interprofessional education and collaboration between general practitioner trainees and practice nurses in providing chronic care; a qualitative study
Source: BMC Med Educ. 2020 Sep 3;20:290. doi: 10.1186/s12909-020-02206-1 (PMC7469346; doi:10.1186/s12909-020-02206-1)
Supplement: Supplementary file 1 — Additional file 1. Final version of the interview guide. [file 12909_2020_2206_MOESM1_ESM.docx]

**Supplementary file:** interview guide (English)

***Introduction***

- introduction of the interview or focus group

- consent for audio recording

- explanation of respondents’ rights

- aim of the study

- duration of the interview or focus group and procedure

- informed consent

***IPE and IPC core questions for GP supervisors, GP trainees and nurse practitioners***

How do you define IPE and IPC?

Tell me about interprofessional activities in your practice?

How do you perceive IPC?

What do you think fosters IPE and IPC?

What do you think challenges or hinders IPC?

As a GP trainee/ nurse practitioner/ GP supervisor working in a GP training practice, what do you think you can learn or benefit from IPC?

As a GP trainee/ nurse practitioner/ GP supervisor working in a GP training practice, what do you think the drawbacks of IPC are?

Can you describe your experiences with the patient-centred communication training?

Does the communication training facilitate how you care for patients or collaborate with GP trainees/practice nurses/ GP supervisor?

Could you give recommendations on how a GP training institute could facilitate interprofessional Collaboration and learning in GP training practice?
